# EPIDEMIOLOGICAL PROFILE OF CONGENITAL FOOT ANOMALIES IN THE STATE OF BAHIA FROM 2014 TO 2022

**DOI:** 10.1590/1413-785220263405e289326

**Published:** 2026-08-10

**Authors:** Jaqueline Nogueira dos Santos, Rafael Pedrosa Braga, Adan Araújo Marques, João Pedro Ferreira Pinho Almeida, Pedro Fernando dos Santos

**Affiliations:** 1 Hospital Regional de Santo Antonio de Jesus (HRSAJ), Santo Antonio de Jesus, BA, Brazil.; 2 Universidade Federal do Recôncavo da Bahia (UFRB), Santo Antonio de Jesus, BA, Brazil.

**Keywords:** Defects, Congenital, Epidemiology, Unified Health System, Anormalidades Congênitas, Epidemiologia, Sistema Único de Saúde

## Abstract

**Objective:**

To investigate the epidemiological profile of births of children with congenital foot abnormalities and their associated factors within the Unified Health System (SUS).

**Methods:**

This study adopts a quantitative, descriptive, and cross-sectional approach, using secondary data from the Live Birth Information System (SINASC), provided by the SUS Department of Informatics (DATASUS). Data from the years 2014 to 2022, related to the State of Bahia, were analyzed. Births with congenital foot malformations were identified through specific ICD-10 codes designating the main diagnosis.

**Results:**

During the period from 2014 to 2022, Bahia recorded a total of 13,347 cases of congenital anomalies, of which 1,364 were related to congenital foot deformities, among a total of 1,765,439 births, representing a rate of 0.77 cases per 1,000 live births.

**Conclusion:**

The presented results indicate that male live births, children of mothers with less than 8 years of education, with a gestational age between 22 and 27 weeks, born by cesarean section, and with Apgar scores of less than 6 in the 1st and 5th minutes, are significantly associated with congenital foot malformations. ***Level of evidence II; Retrospective study.***

## RESUMO

**Objetivo:**

Investigar o perfil epidemiológico de nascimentos de crianças com Anormalidades Congênitas do Pé e seus fatores associados no âmbito do Sistema Único de Saúde (SUS).

**Métodos:**

Este estudo adota uma abordagem quantitativa, descritiva e transversal, utilizando dados secundários provenientes do Sistema de Informação sobre Nascidos Vivos (SINASC), disponibilizados pelo Departamento de Informática do SUS (DATASUS). Foram analisados os dados referentes aos anos de 2014 a 2022, referentes ao Estado da Bahia. Os nascimentos com Malformações Congênitas do pé foram identificados através de códigos específicos da CID-10 que designam o diagnóstico principal.

**Resultados:**

Durante o período de 2014 a 2022, a Bahia registrou um total de 13.347 casos de anomalias congênitas, dos quais 1.364 foram relacionados a deformidades congênitas nos pés, em meio a um universo de 1.765.439 nascimentos, representando uma taxa de 0,77 casos por cada 1.000 nascidos vivos.

**Conclusão:**

Os resultados apresentados indicam que nascidos vivos do sexo masculino, filhos de mães com escolaridade inferior a 8 anos, com idade gestacional entre 22 e 27 semanas, nascidos por cesariana e com Apgar no 1º e 5º minutos menor que 6 estão significativamente associados a malformações congênitas do pé. ***Nível de evidência II; Estudo retrospectivo.***

## INTRODUCTION

Congenital malformations are morphological defects present at birth that can affect the structure of organs or systems, varying widely in severity and complexity. Such defects can significantly impact the functionality and quality of life of the affected individual^1^. Among the etiologies of congenital foot deformities are inadequate movement during the gestational period, idiopathic, neurogenic, syndromic, intrinsic, and extrinsic causes, the latter being the most common^2^. The gestational period most vulnerable for limb development, due to its complexity and exposure to the uterine wall, lies between the 24th and 36th day post-fertilization, this interval being critical for the influence of environmental factors. The diagnosis of these conditions can be made during prenatal care, at birth, or through physical examination of the newborn^3^.

In the country, data on the prevalence and incidence of Congenital Foot Anomalies in their different types are still underestimated. Currently, it is known that these defects, together with congenital malformations of the musculoskeletal system, account for approximately 50 of all congenital deformities, since they are more easily detected during physical examination at birth^4^. There is greater emphasis on information about congenital clubfoot or talipes equinovarus, characterized by a deformity of the foot and ankle region, which may include cavus, varus, equinus, and adduction^5^, which is one of the most common congenital deformities, affecting 1 case per 1,000 live births^6^.

On the other hand, the Live Birth Information System (SINASC) provides high-quality data with national coverage^7,8^, enabling the development of various investigations into perinatal care.

In the state of Bahia, which presents significant disparities in socioeconomic, demographic, and health indicators^9^, there is a favorable setting for conducting relevant studies on congenital foot deformities. Understanding the epidemiological profile of these deformities is essential to improve prenatal diagnosis and implement early and effective therapeutic interventions, aiming at improving children's quality of life.

Therefore, this study aims to investigate the epidemiological profile of births of children with Congenital Foot Anomalies and their associated factors within the Unified Health System (SUS).

## METHODS

### Study design and context

This is a quantitative epidemiological, cross-sectional study with individual secondary data from SINASC through DATASUS, extracted in 2024. The data refer to the prevalence of births with Congenital Foot Anomalies among live births in the State of Bahia between 2014 and 2022. This period was chosen because, at the time of extraction, no information was available for dates after 2022. This state has a territorial area of 567,295 km^2^ divided into 417 municipalities with 9 health macro-regions. In 2022, the estimated number of live births was 171,272.

### Study population

The study population consisted of individuals born alive between January 2014 and December 2022 who were registered in SINASC. The following congenital foot deformities were included in the study, classified according to codes within the range (Q660–Q669) of the Live Birth Declaration form, which contains the list of congenital malformation or anomaly codes, coded using the International Statistical Classification of Diseases and Related Health Problems, 10th revision (ICD-10). Cases with incomplete or inconclusive data were excluded.

### Data collection instrument

The database used is publicly available; data are collected and processed by the Ministry of Health, therefore personal identification data were not included. The information repository has a high coverage of live birth information, representing more than 90% of births occurring in the country^7^. The Live Birth Declaration form (DNV) is the basic data collection instrument, which must be mandatorily completed by healthcare professionals responsible for attending the newborn during the birth period. Data are extracted from hospital maternity medical records and entered into the electronic system by trained staff.

### Other covariates investigated

Among the variables applied, sociodemographic, economic, and clinical history characteristics of probable factors associated with congenital foot deformities were included, as reported through the DNV. The following variables were considered: place of birth occurrence (hospital, home, other establishment); maternal age (in years), marital status (with partner/without partner), level of education (none, primary school, higher education, unknown), mother's health region of residence, type of delivery (vaginal or cesarean), sex (male or female), Apgar score at the first and fifth minute, race/color (Black and non-Black), weeks of gestation, type of pregnancy.

### Statistical analysis

The prevalence of Congenital Foot Anomaly cases among live births was determined through descriptive statistics, obtaining simple and relative frequencies, as well as the chi-square test χ 2 to assess the association of categorical variables of interest, with a statistically significant level set at p-values less than 0.05. The prevalence ratio (PR) and its respective 95% confidence interval (95% CI) were also obtained for comparisons between the described variables of interest and Congenital Foot Anomaly cases. For this purpose, the statistical software STATA (Data Analysis and Statistical Software) was used.

## RESULTS

During the period from 2018 to 2022, Bahia recorded a total of 13,347 cases of congenital anomalies, of which 1,364 were related to congenital foot deformities, among a total of 1,765,439 births, representing a rate of 0.77 cases per 1,000 live births.

When examining the socioeconomic-demographic characteristics of live births in the bivariate analysis, the highest prevalences of congenital foot anomalies were observed among male individuals, with 1,006 cases (1.11/1,000 live births) PR 1.42, 95% CI (1.29; 1.47); non-Black individuals in terms of race/color, with 161 cases (1.08/1,000 live births) PR 1.10, 95% CI (0.93; 1.29); those born in other establishments (neither hospitals nor other healthcare facilities), with 6 cases (1.34/1,000 live births) PR 1.40, 95% CI (0.62; 3.11); those born by cesarean section, with 872 cases (1.10/1,000 live births) PR 1.30, 95% CI (1.19; 1.44); those with 1st-minute Apgar < 06, with 281 cases (3.95/1,000 live births) PR 4.48, 95% CI (1.12; 5.33); and 5th-minute Apgar also < 06, with 108 cases (6.92/1,000 live births) PR 7.52, 95% CI (6.19; 9.14), as shown in Table 1.

**Table 1:** Prevalence of congenital foot malformations according to sociodemographic and obstetric characteristics. Bahia, January 2014 to December 2022. (n=1364)

Variables	n (prevalence/1,000 live births)	PR (95% CI)	P-Value
**Sex**			
Male	1,006 (1.11)	1.42 (1.29;1.57)	<0.05
Female	675 (0.78)		
**Race/color**			
Black	1,475 (0.98)		
Non-Black	161 (1.08)	1.10 (0.93; 1.29)	>0.05
**Mother's race/color**			
Black	1,455 (0.98)		
Non-Black	161 (1.10)	1.12 (0.95;1.32)	>0.05
**Place of birth occurrence**			
Hospital	1,627 (0.96)	0.98 (0.70;1.14)	>0.05
Other healthcare facility	51 (1.04)	1.09 (0.82; 1.44)	>0.05
Home	9 (1.03)	1.07 (0.56; 2.06)	>0.05
Other	6 (1.34)	1.40 (0.62; 3.11)	>0.05
**Mother's marital status**			
With partner	820 (0.92)		
Without partner	830 (1.00)	1.08 (0.98; 1.19)	>0.05
Mother's education (years)*			
≥8	430 (0.34)		
<8	1,225 (2.91)	8.64 (7.44; 9.64)	< 0.05
**Weeks of gestation**			
< 22 weeks	4 (2.86)	2.78 (1.04;7.41)	< 0.05
22 to 27 weeks	30 (3.11)	3.04 (2.14; 4.39)	< 0.05
28 to 31 weeks	49 (2.63)	2.60 (1.96; 3.50)	< 0.05
32 to 36 weeks	270 (1.70)	1.77 (1.55;2.01)	< 0.05
37 to 41 weeks	1,332 (0.92)	0.57 (0.51;0.64)	< 0.05
≥42 weeks	58 (0.97)	0.84 (0.65; 1.09)	
**Type of pregnancy**			
Single	1,639 (0.95)		
Twin	45 (1.26)	1.33 (0.99; 1.78)	>0.05
**Type of delivery**			
Vaginal	815 (0.84)		
Cesarean	872 (1.10)	1.30 (1.19;1.44)	<0.05
**Apgar 1**			
< 6	281 (3.95)	4.48 (1.12; 5.33)	<0.05
≥ 6	1,362 (0.84)		
**Apgar 5**			
< 6	108 (6.92)	7.52 (6.19;9.14)	<0.05
≥ 6	1,535 (0.92)		

Source: Live Birth Information System (SINASC), 2022.

Furthermore, when examining the prevalence of congenital foot deformities according to maternal and gestational characteristics, the highest records were noted among individuals whose mothers had no partner, with 830 cases (1.00/1,000 live births) PR 1.08, 95% CI (0.98; 1.19); with less than 08 years of schooling, with 1,225 cases (2.91/1,000 live births) PR 8.64, 95% CI (7.44; 9.64); with gestational age of 22 to 27 weeks, with 30 cases (3.11/1,000 live births) PR 2.04, 95% CI (2.14; 4.39); and with twin pregnancy, with 45 cases (1.26/1,000 live births) PR 1.33, 95% CI (0.99; 1.78), as illustrated in Table 1.

Among congenital foot anomalies, the most prevalent were other deformities, totaling 687 cases (40.5/1,000 live births), followed by unspecified deformities, with 259 cases (15.3/1,000 live births). Subsequently, 217 cases (12.8/1,000 live births) of clubfoot calcaneovalgus and 215 cases (12.7/1,000 live births) of clubfoot equinovarus were observed, as indicated in Table 2.

**Table 2:** Distribution of the prevalence of Congenital Foot Deformities, according to the International Statistical Classification of Diseases and Related Health Problems, 10th revision (ICD-10). Bahia, January 2014 to December 2022.

Congenital foot anomaly	n(%)
Q660 - Clubfoot equinovarus	215 (12.7)
Q661 - Clubfoot calcaneovarus	103 (6.1)
Q662 - Metatarsus varus	-
Q663 - Other congenital varus deformities of the feet	45 (2.7)
Q664 - Clubfoot calcaneovalgus	217 (12.8)
Q665 - Congenital flat foot	106 (6.3)
Q666 - Other congenital valgus deformities of the feet	45 (2.7)
Q667 - Pes cavus	5 (0.3)
Q668 - Other congenital deformities of the foot	687 (40.5)
Q669 - Congenital deformity of the foot, unspecified	259 (15.3)

Source: Live Birth Information System (SINASC), 2022.

## DISCUSSION

The prevalence of congenital foot deformities found in the present study was 0.77 cases per 1,000 live births, a coefficient slightly lower than that obtained in the investigation of Congenital Clubfoot in a population-based study in European countries, which was 0.92 cases per 1,000 live births^10^. Furthermore, it is lower than the prevalence of 2 cases per 1,000 live births observed in research covering the entire national territory^11^. These data do not rule out the hypothesis of persistent underreporting in the Live Birth Declaration (DNV) field, even after comparing the validity of congenital defect cases reported in the Live Birth Information System in Brazil and the observed improvement in SINASC data quality, as rates still fall below the actual figures^7,12^.

In this study, higher prevalences of congenital foot malformations were found among male individuals, with a rate of 1.11 per 1,000 live births. These results corroborate findings from previous research on all congenital malformations, highlighting the need for a more in-depth examination of the intrinsic (genetic) factors that influence this population^13^. The association between congenital foot deformities and cesarean deliveries, evidenced in other studies, suggests a preferential medical indication for cesarean section following a diagnosis of congenital malformation. This scenario is heightened by the high rate of cesarean deliveries performed in the state capital, which exceeds the averages of the other states in the country^14,15,16^.

Among adverse birth outcomes, a significant association was observed between congenital foot anomaly and unsatisfactory Apgar scores at the first and fifth minute, as well as prematurity, as demonstrated in studies using different methodological approaches conducted in the country^17^. Another hypothesis for this finding is based on the condition that individuals subjected to this exposure are allocated to higher-complexity care units, such as the Intensive Care Unit, where they receive more detailed investigation and, consequently, are less underdiagnosed^18^.

The slight increase in congenital foot defect coefficients among children of mothers without partners and the high prevalence among children of mothers with low levels of education point to a possible association between socioeconomic factors and the outcome in question. This is because mothers with less social and educational support may be exposed to additional adversities during the gestational period, such as reduced access to health services and inadequate, low-quality prenatal care^19,20^, which have been considered factors associated with congenital deformities^21^.

In contrast, Black live births and children of Black mothers had a lower prevalence of the outcome in question, but this was not a conclusive association. This does not rule out the aforementioned hypothesis, as Black pregnant women also face vulnerabilities regarding access to health services^22^.

Furthermore, the higher coefficients among twin pregnancies and congenital foot deformities, and among those born in establishments other than home or healthcare facilities, suggest a risk factor, though not a significant one.

Despite the limitations of this study, such as the cross-sectional design and the use of publicly available secondary data, which restrict the generalization of the results, this investigation is relevant for highlighting the epidemiological distribution of congenital foot malformation cases and their associated factors, a context that is little explored in countries such as Brazil. Furthermore, for interpreting the results, it is unnecessary to consider the aggregate effect known as the ecological fallacy, since the statistical analyses were performed on individual-level data, reducing distortions in the findings.

Therefore, it is suggested that more robust investigations on the subject be conducted, especially regarding sociodemographic, gestational, neonatal, and modifiable predictive factors. Such studies could identify preventable factors that deserve greater attention from healthcare professionals, management teams, and the families of affected individuals.

## CONCLUSION

The presented results indicate that male live births, children of mothers with less than 8 years of education, with a gestational age between 22 and 27 weeks, born by cesarean section, and with Apgar scores of less than 6 in the 1st and 5th minutes, are significantly associated with congenital foot malformations. However, these data should be interpreted with caution, as they come from publicly available secondary sources and reflect the resource limitations typical of regions in northeastern Brazil. Although this study is specific to the State of Bahia and has its respective limitations, it is believed that the conclusions may be applicable to states with similar population profiles.

These data highlight the importance of interventions directed at specific risk groups for the prevention and early detection of congenital malformations. Furthermore, they underscore the need for public policies aimed at improving maternal education and ensuring adequate prenatal follow-up, especially in high-risk pregnancies, in order to mitigate the extrinsic factors associated with the development of these conditions.

## Data Availability

The contents underlying the research text are available upon reviewers’ request.

## References

[1] Motta GR, Barros TEP (2018). Ortopedia e traumatologia.

[2] São Paulo. Prefeitura Municipal (2012). Secretaria Municipal da Saúde. Manual de aperfeiçoamento no diagnóstico de anomalias congênitas.

[3] Brasil. Ministério da Saúde. Secretaria de Vigilância em Saúde. Departamento de Análise em Saúde e Vigilância de Doenças Não Transmissíveis (2021). Saúde Brasil 2020/2021: anomalias congênitas prioritárias para vigilância ao nascimento.

[4] Lima NA, Lucena EES, Araújo NM, Oliveira TC, Silva CF (2018). Perfil epidemiológico das malformações congênitas em recém-nascidos no estado do Rio Grande do Norte no período de 2004 a 2011. Rev Bras Cienc Saude..

[5] Magriples U, Wilkins-Haug L, Barss VA (2023). UpToDate.

[6] Maranho DAC, Volpon JB (2011). Pé torto congênito. Acta Ortop Bras..

[7] Szwarcwald CL, Leal MC, Esteves-Pereira AP, Almeida WS, Frias PG, Damacena GN (2019). Avaliação das informações do Sistema de Informações sobre Nascidos Vivos (SINASC), Brasil. Cad Saude Publica..

[8] Theme Filha MM, Gama SGN, Cunha CB, Leal MC (2004). Confiabilidade do Sistema de Informações sobre Nascidos Vivos hospitalares no município do Rio de Janeiro, 1999-2001. Cad Saude Publica.

[9] Goes EF, Nascimento ER (2013). Mulheres negras e brancas e os níveis de acesso aos serviços preventivos de saúde: uma análise sobre as desigualdades. Saude Debate..

[10] Wang H, Barisic I, Loane M, Addor MC, Garne E, Morris JK (2019). Clubfoot in Europe: a population-based study. Am J Med Genet A..

[11] Brasil. Ministério da Saúde (2023). Política Nacional de Saúde da Pessoa com Deficiência: Política Nacional Pé Torto Congênito.

[12] De Nicola PDR, Cernach MCSP, Perez ABA, Brunoni D (2010). A utilização da Internet na notificação dos defeitos congênitos na Declaração de Nascido Vivo em quatro maternidades públicas do município de São Paulo, Brasil. Cad Saude Publica..

[13] Mendes CQS, Avena MJ, Mandetta MA, Balieiro MMFG (2015). Prevalência de nascidos vivos com anomalias congênitas no município de São Paulo. Rev Soc Bras Enferm Ped..

[14] Domingues RMSM, Dias MAB, Pereira MN, Torres JA, d’Orsi E, Pereira APE (2014). Processo de decisão pelo tipo de parto no Brasil: da preferência inicial das mulheres à via de parto final. Cad Saude Publica.

[15] Pinto CO, Nascimento LFC (2007). Estudo de prevalência de defeitos congênitos no Vale do Paraíba Paulista. Rev Paul Pediatr..

[16] Pinto EP, Luz LA, Guimarães MAP, Tavares LT, Brito TRS, Souza GDF (2017). Prevalência e fatores associados às anomalias congênitas em recém-nascidos. Rev Bras Promoc Saude..

[17] Nhoncanse GC, Germano CMR, Avó LRS, Melo DG (2014). Aspectos maternos e perinatais dos defeitos congênitos: um estudo caso-controle. Rev Paul Pediatr..

[18] Nascimento LFC (2008). Prevalência de defeitos de fechamento de tubo neural no Vale do Paraíba, São Paulo. Rev Paul Pediatr..

[19] Wang H, Frasco E, Takesue R, Tang K (2021;). Maternal education level and maternal healthcare utilization in the Democratic Republic of the Congo: an analysis of the Multiple Indicator Cluster Survey 2017/18. BMC Health Serv Res..

[20] Bintabara D, Mwampagatwa I (2023). Socioeconomic inequalities in maternal healthcare utilization: an analysis of the interaction between wealth status and education in Tanzania. PLOS Glob Public Health..

[21] Mashuda F, Zuechner A, Chalya PL, Kidenya BR, Manyama M (2014;). Pattern and factors associated with congenital anomalies among young infants admitted at Bugando Medical Centre, Mwanza, Tanzania. BMC Res Notes..

[22] Lessa MSAA, Nascimento ER, Coelho EAC, Soares IJ, Rodrigues QP, Santos CAST (2022). Pré-natal da mulher brasileira: desigualdades raciais e suas implicações para o cuidado. Cien Saude Colet..

